# Supplementary material for: Collagen VI Is a Gi-Biased Ligand of the Adhesion GPCR GPR126/ADGRG6
Source: Cells. 2023 Jun 5;12(11):1551. doi: 10.3390/cells12111551 (PMC10252604; doi:10.3390/cells12111551)
Supplement: Supplementary file 1 [file cells-12-01551-s001.zip › cells-2332993-supplementary.pdf]

Supplemental Table S1. Summary of GPR126 446-807 domain specific pulldown partners from sciatic nerve.

Peptide number	Gene name	Protein description	localization
**18**	HS90B	**Heat shock protein HSP 90-beta**	cytoplasm
**10**	IgG1	**Ig heavy chain V region**	
**10**	ENPL	**Endoplasmin**	Endoplasmic reticulum
**8**	KPYM	**Pyruvate kinase PKM**	Cytoplasm<br>Nucleus
**8**	FUT8	**Alpha-(1,6)-fucosyltransferase**	Golgi
**5**	HS90A	**Heat shock protein HSP 90-alpha**	cytoplasm
**5**	IgG2a	**Ig gamma-2A chain C region**	
**5**	NID2	**Nidogen-2**	Basement membrane<br>Extracellular matrix<br>Secreted
**5**	Q9Z1A1	Evidence at transcript level	
**4**	GPR126	G protein coupled receptor 126	membrane
**4**	EF2	**Elongation factor 2**	nucleus
**4**	FINC	**Fibronectin**	Extracellular matrix<br>Secreted
**4**	FBLN5	**Fibulin-5**	Secreted
**4**	SERA	**D-3-phosphoglycerate dehydrogenase**	
**4**	ACLY	Acly protein	mitochondrion
**4**	TCPA	**T-complex protein 1 subunit alpha**	Cytoplasm<br>Cytoskeleton
**3**	ACTS	**Actin, alpha skeletal muscle**	Cytoplasm<br>Cytoskeleton
**3**	LAMA2	**Laminin subunit alpha-2**	Basement membrane<br>Extracellular matrix<br>Secreted
**3**	CO6A1	**Collagen alpha-1(VI) chain**	Extracellular matrix<br>Secreted
**3**	CN37	**2',3'-cyclic-nucleotide 3'-phosphodiesterase**	Membrane
**3**	PCNA	**Proliferating cell nuclear antigen**	Nucleus
**3**	ECHA	**Trifunctional enzyme subunit alpha, mitochondrial**	mitochondrial
**3**	BACH	**Transcription regulator protein BACH1**	Nucleus
**3**	NONO	**Non-POU domain-containing octamer-binding protein**	Nucleus

Protocol for sciatic nerve co-IP:

Step I: Cell Lysate Preparation:

1. collect the SN in an Eppendorf and with a plastic pestle (http://www.fishersci.com/ecomm/servlet/fsproductdetail_10652_617322__-1_0) on liquid nitrogen to reduce them as a powder.
2. add 300ul of ice cold RAPI Buffer containing protease inhibitors, lyse cells with a cell disruptor for 10' and homogenization with syringe needle 26 G.
3.. Centrifuge the cell lysate at 10,000xg for 10minutes at 4°C.
3. Carefully collect the supernatant without disturbing the pelletand transfer to a clean tube. The Pellet can be discarded
4.The protein concentration can be determined by Bradford assay.

Step II: Cell Lysate Preclearing

1. preclearing cell lysate with 100ul protein G beads (from Invitrogen) equilibrated in the corresponding IP buffer/Lysis Buffer.

Step III: Immunoprecipitation

1. Incubation 250ug (in 300ul) of precleared cell lysate 3 hours with 2ug of Fc or Gpr126-hFc recombinant proteins followed by 1h with 40 ul of protein G sepharose beads (GE Healthcare 17-0618-01)
2.Centrifuge the tube at 2,500xg for 30 seconds at 4°C.
3.Carefully discard the supernatant completely. Wash the beads four times with 900µLof ice-cold Lysis Buffer, one time with TBS buffer. Centrifuge to pellet the beads in between each wash. In order to minimize background, remove the supernatant completely after each wash.
5.After the last wash, carefully aspirate the supernatant and add 50µL 1X Laemmli loading buffer to the bead pellet.
5. Vortex and heat at 90-100°C for 5 minutes.
6. Centrifugeat 10,000xg for 1 minutes to pellet the beads. Collect the supernatant carefully and load onto an SDS-PAGE gel.
7. Transfer to membrane, followed by western blot analysis.

Protocol for sciatic nerve lysing and GPR126-mFc-biotag pull- down:

Step I: Cell Lysate Preparation:

1. collect the SN in an Eppendorf and with a plastic pestle (http://www.fishersci.com/ecomm/servlet/fsproductdetail_10652_617322__-1_0) on liquid nitrogen to reduce them as a powder.
2. add 300ul of ice cold RAPI Buffer containing protease inhibitors, lyse cells with a cell disruptor for 10' and homogenization with syringe needle 26 G.
3.. Centrifuge the cell lysate at 10,000xg for 10minutes at 4°C.
3. Carefully collect the supernatant without disturbing the pelletand transfer to a clean tube. The Pellet can be discarded
4.The protein concentration can be determined by Bradford assay.

Step II: Cell Lysate Preclearing

1. preclearing cell lysate with 300ul streptavidin beads, equilibrated in the corresponding IP buffer/Lysis Buffer.

Step III: in vitro biotinylation

1 part Biotin: 1ul
1 part Biomix A: 1ul
1 part Biomix B: 1ul
6 parts substrate solution: 6ul (1ug/ul)
BirA enzyme 1 ul (1ug/ul)
Incubate at RM for overnight

BirA Biotin-Protein Ligase Standard Reaction Kit	Cat. BIRA-500	MSPP-BIRA500

Delivery information: Standard Reaction Kit includes: 2 vials of 20 µl each 1mg/ml BirA biotin-protein ligase (40 µg total); 2 vials of 1.5 ml each BiomixA (0.5 M bicine buffer solution, pH 8.3); 2 vials of 1.5 ml each BiomixB (100 mM ATP, 100 mM Mg(OAc)2, 500 µM d-biotin); 2 vials of 1.5 ml each additional BIO-200 (500 µM d-biotin).

Step IV: Immunoprecipitation

1. Incubation precleared cell lysate 3 hours with 4ug of in vitro biotinylated **GPR56-mFc-biotag protein, or mFc-biotag as negative control,** followed by 1h with 100 ul of Streptavidin beads (Sigma)
2.Centrifuge the tube at 2,500xg for 30 seconds at 4°C.
3.Carefully remove the supernatant completely. Wash the beads four times with 900µLof ice-cold Lysis Buffer, one time (final wash) with TBS buffer. Centrifuge to pellet the beads in between each wash. In order to minimize background, care should be given to remove the supernatant completely after each wash.
5.After the last wash, carefully aspirate the supernatant and add 60µLof 1X Laemmli sample buffer to the bead pellet.
5. Vortex and heat at 90-100°C for 5 minutes.

6. Centrifugeat 10,000xg for 1 minutes to pellet the beads.Collect the supernatant carefully and load onto an SDS-PAGE gel. The whole lane were excised and submitted to Taplin Biological Mass Spectrometry Facility, Harvard Medical Schoo.

Step V: Protein Sequence Analysis by LC-MS/MS
At Taplin Biological Mass Spectrometry Facility, Harvard Medical School

The gels were cut into approximately 1 mm^3 pieces. Gel pieces were then subjected to a modified in-gel trypsin digestion procedure (1). Gel pieces were washed and dehydrated with acetonitrile for 10 min. followed by removal of acetonitrile. Pieces were then completely dried in a speed-vac. Rehydration of the gel pieces was with 50 mM ammonium bicarbonate solution containing 12.5 ng/µl modified sequencing-grade trypsin (Promega, Madison, WI) at 4ºC. After 45 min., the excess trypsin solution was removed and replaced with 50 mM ammonium bicarbonate solution to just cover the gel pieces. Samples were then placed in a 37ºC room overnight. Peptides were later extracted by removing the ammonium bicarbonate solution, followed by one wash with a solution containing 50% acetonitrile and 1% formic acid. The extracts were then dried in a speed-vac (~1 hr). The samples were then stored at 4ºC until analysis.

On the day of analysis the samples were reconstituted in 5 - 10 µl of HPLC solvent A (2.5% acetonitrile, 0.1% formic acid). A nano-scale reverse-phase HPLC capillary column was created by packing 2.6 µm C18 spherical silica beads into a fused silica capillary (100 µm inner diameter x ~30 cm length) with a flame-drawn tip (2). After equilibrating the column each sample was loaded via a Famos auto sampler (LC Packings, San Francisco CA) onto the column. A gradient was formed and peptides were eluted with increasing concentrations of solvent B (97.5% acetonitrile, 0.1% formic acid).

As peptides eluted they were subjected to electrospray ionization and then entered into an LTQ Orbitrap Velos Pro ion-trap mass spectrometer (Thermo Fisher Scientific, Waltham, MA). Peptides were detected, isolated, and fragmented to produce a tandem mass spectrum of specific fragment ions for each peptide. Peptide sequences (and hence protein identity) were determined by matching protein databases with the acquired fragmentation pattern by the software program, Sequest (Thermo Fisher Scientific, Waltham, MA) (3). All databases include a reversed version of all the sequences.

1. Shevchenko, A.; Wilm, M.; Vorm, O.; Mann, M. Mass spectrometric sequencing of proteins silver-stained polyacrylamide gels. Anal Chem. 1996, 68, 850–858. https://doi: 10.1021/ac950914h.
2. Peng, J.; Gygi, S.P. Proteomics: The move to mixtures. J. Mass Spec. 2001, 36, 1083–1091. https://doi: 10.1002/jms.229.
3. Eng, J.K.; McCormack, A.L.; Yates, J.R. An approach to correlate tandem mass spectral data of peptides with amino acid sequences in a protein database. J Am Soc Mass Spectrom. 1994, 5, 976–989. https://doi: 10.1016/1044-0305(94)80016-2.

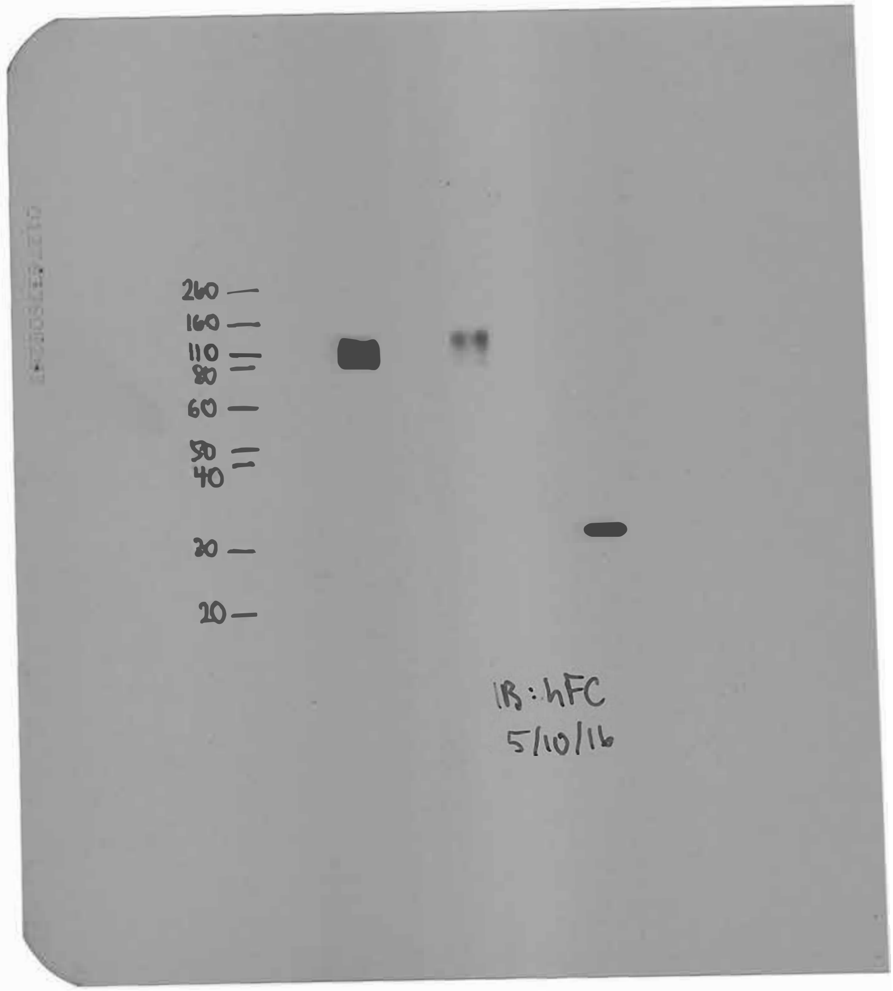
260
160
110
80
60
50
40
30
20
IB: hFC
5/10/16

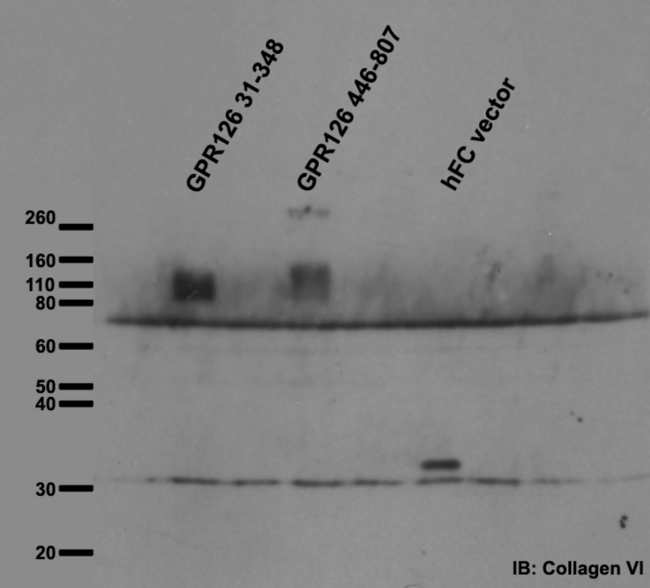
GPR126 31-348
GPR126 446-807
hFC vector
260
160
110
80
60
50
40
30
20
IB: Collagen VI
